# Genes Upregulated in Winter Wheat (*Triticum aestivum* L.) during Mild Freezing and Subsequent Thawing Suggest Sequential Activation of Multiple Response Mechanisms

**DOI:** 10.1371/journal.pone.0133166

**Published:** 2015-07-14

**Authors:** Daniel Z. Skinner

**Affiliations:** USDA-ARS and Washington State University, Department of Crop and Soil Sciences, 209 Johnson Hall, Pullman, WA, 99164, United States of America; Huazhong University of Science and Technology, CHINA

## Abstract

Exposing fully cold-acclimated wheat plants to a mild freeze-thaw cycle of −3°C for 24h followed by +3°C for 24 or 48h results in dramatically improved tolerance of subsequent exposure to sub-freezing temperatures. Gene enrichment analysis of crown tissue from plants collected before or after the −3°C freeze or after thawing at +3°C for 24 or 48h revealed that many biological processes and molecular functions were activated during the freeze-thaw cycle in an increasing cascade of responses such that over 150 processes or functions were significantly enhanced by the end of the 48 h, post-freeze thaw. Nearly 2,000 individual genes were upregulated more than 2-fold over the 72 h course of freezing and thawing, but more than 70% of these genes were upregulated during only one of the time periods examined, suggesting a series of genes and gene functions were involved in activation of the processes that led to enhanced freezing tolerance. This series of functions appeared to include extensive cell signaling, activation of stress response mechanisms and the phenylpropanoid biosynthetic pathway, extensive modification of secondary metabolites, and physical restructuring of cell membranes. By identifying plant lines that are especially able to activate these multiple mechanisms it may be possible to develop lines with enhanced winterhardiness.

## Introduction

The emerging picture of the transcriptomic response of winter wheat (*Triticum aestivum* L, AABBDD, 2n = 6x = 42) plants to cold temperature indicates a very complex process that differs according to the kind of cold stress applied and the genotype of the plants studied. Several studies have reported that hundreds of genes in wheat respond to the onset of cold temperatures [1–3] and that there are large differences in expression levels in leaf tissue compared to crown tissue [4–8]. It was reported [9] that over 12,000 genes in wheat changed expression during 0–70 days of exposure to 6°C, suggesting that “at least two regulatory networks control the level of expression of many of the known cold-responsive genes.” Herman et al. [10] demonstrated that the ability of winter wheat plants, acclimated for three weeks at +3° C, to survive subsequent exposure to potentially damaging temperatures was greatly improved by exposure to −3°C for several hours, a process the authors referred to as “subzero acclimation.” This freezing tolerance improvement was accompanied by numerous transcriptomic, physiological, and metabolic changes, leading the authors to suggest that the additional freezing tolerance was the result of "complex biological processes" [10]. Skinner and Bellinger [11] demonstrated that exposure of fully cold-acclimated plants to −3°C for 16 h led to significantly improved tolerance of subsequent freezing to potentially damaging temperatures, and that allowing the plants to thaw for 24 hours at a cold acclimation-inducing temperature after the −3°C freeze led to even greater survival. These results suggested that biological processes initiated during exposure to −3°C continued to develop and possibly diversify during a subsequent thaw at low, above-freezing temperature, resulting in a further increase in freezing tolerance.

Winter wheat plants in the field frequently are exposed to mild freeze-thaw events similar to those studied by Skinner and Bellinger [11] when nighttime temperatures fall below freezing followed by above-freezing temperatures the next day as the autumn season transitions to winter and sustained subzero temperatures. It seems reasonable to suppose that wheat plants have developed an adaptive response to these mild freeze-thaw events. By understanding the processes involved in the development of the freeze-thaw enhanced freezing tolerance we have observed [11], it may be possible to identify plant lines especially effective in the development of the response with subsequent improved winterhardiness. Accordingly, the objective of this study was to identify the biological processes and genes involved in the response of cold-acclimated plants to a −3°C freeze, and the genes involved in response to a subsequent thaw at +3°C, with a view to identifying key pathways that can be evaluated for the purpose of identifying wheat lines that are especially competent in effecting parts of freeze-thaw enhanced freezing tolerance. Such lines would be expected to provide sources of improved freezing tolerance and winter hardiness.

## Materials and Methods

### Plant Material and freezing treatment

Transcriptomic changes were studied in the hard red winter wheat cultivar Tiber, a selection from Redwin [12]. The LT_50_, the temperature expected to be fatal to 50% of the plants, is about −13°C for Tiber [13].

Seeds were sown into Sunshine Mix LC1 planting medium (Sun Gro Horticulture, Bellevue, WA, USA) in 6-container packs (Model 1020, Blackmore Co., Belleville, MI, USA). Seeds were germinated and seedlings grown at 22°C in a growth chamber (Model E15, Conviron, Pembina, ND) under cool white fluorescent lights (about 300 μmol m^-2^ s^-1^ at the soil surface) with a 16 h photoperiod until the seedlings reached the three-leaf stage. The plants were then acclimated to low temperature at 3°C with a 12-hour (h) photoperiod (about 250 μmol m^-2^ s^-1^ at mid-plant height) for 4 weeks (wks). Plants were irrigated weekly with nutrient solution containing macro and micronutrients (Peters Professional, Scotts Co., Camarillo, CA, USA).

Transcriptomic changes were assessed in the crowns (meristematic regions) of the plants after three temperature treatments and the control. The control treatment was represented by plants that had been acclimated at 3°C with a 12 h photoperiod for 4 wks. For the first temperature treatment, designated the subzero acclimation (SZA) treatment, the crowns of the plants were exposed by gently washing the soil from the tops of the plant packs with ice water; care was taken to disturb the plants and their roots as little as possible, then these plant packs were placed in a freezer at −3°C for 24 h. The plants to be assayed for the subzero acclimation treatment were then quickly removed from the top of the soil and dropped into liquid nitrogen. Using forceps, the crown regions were separated from the rest of the plant and stored at −80°C prior to RNA extraction. For the remaining two temperature treatments, plants were assayed after 24 or 48 h of thawing at +3°C after they had been frozen to −3°C for 24 h as described above. These plants were removed from the −3°C freezer, placed in an incubator at +3°C, and allowed to remain for the designated time. The plants were then removed and dropped in liquid nitrogen and treated as above.

### RNA extraction, microarray interrogation, and qPCR

To extract RNA, six crowns were homogenized in liquid nitrogen using a mortar and pestle; RNA was extracted using 1.5ml of Trizol reagent and the standard protocol (Invitrogen, Carlsbad, CA). RNA was quantified with UV spectrophotometry and stored at −80°C. About 40μg RNA were recovered from six plant crowns.

Global gene expression levels were assayed with the GeneChip Wheat Genome Array (Affymetrix, Santa Clara, CA). This array contains 61,127 probe sets representing 55,052 transcripts from 20,314 unigenes from all 42 wheat chromosomes (http://www.affymetrix.com, Data sheet for GeneChip Wheat Genome Array, Part number 701797, Rev. 2).

Three to five completely independent replications (plants grown at different times and processed separately) were analyzed from each treatment. RNA labeling and hybridization to the Affymetrix arrays, and post-hybridization scanning and data pre-processing was conducted by the Washington State University Biotechnology Core Facility. The data files were further analyzed using "Flexarray" software [14]. The microarray data were normalized using the "Robust Multi-chip Average" (RMA) method and probe sets showing significant differences in hybridization intensity among treatments were sought using the empirical Bayes analysis (algorithm EB Wright & Simon) available in the Flexarray software. Fold change was calculated by the Flexarray software as 2 raised to the power of the base 2 logarithm of the difference in mean expression of the treatments compared [14].

Fold change was used to illustrate changes in gene expression, but it has been shown that fold-change frequently under-represents the range of genes that responded to a treatment [15], Thus, the numbers of up- or down-regulated genes indicated by fold-change should be considered as the minima. The “characteristic direction” approach appears to provide a more comprehensive indication of transcriptomic response, and a useful overview of expression regulation can be obtained by examining a set of the most strongly regulated genes, such as the most strongly regulated 500, 1000, or 2000 genes [15]. Accordingly, in this study, a gene enrichment analysis based on gene ontology (GO) [16] was undertaken including the 1000 most strongly upregulated, and the 1000 most strongly down-regulated probesets from each of the temperature treatments, as indicated by the “characteristic direction” [15] measurement. The gene ontology assignments were found using the online analysis tool and the GO annotations of the Affymetrix Wheat GeneChip provided with the “Agrigo” project [17]. Gene ontology figures were drawn using the OBO-Edit image editor [18].

To examine expression level changes of individual genes, in addition to the GO term annotations, the GenBank (http://blast.ncbi.nlm.nih.gov) identifiers associated with the microarray probe sets were used to seek tentative functions of genes of interest through BLASTN searches of the GenBank nonredundant database. In the event the BLASTN algorithm returned only genes of unknown function, translated searches (BLASTX and TBLASTX) were also performed in an attempt to increase the number of identified genes. These results were used to classify individual genes into broad functional categories to facilitate evaluation of transcriptomic responses from a gene function perspective.

Quantitative real-time PCR (qPCR) was used to confirm expression dynamics of selected genes. The Protoscript II kit (New England Biolabs, Ipswich, MA, USA) was used to synthesize complementary DNA (cDNA). The cDNA synthesis reactions were initiated with 1 μg RNA that had been treated with RNase-free DNase I (Ambion, Austin, TX, USA) according to the manufacturer’s protocol and were primed with the anchored oligo dT primers provided in the Protoscript II kit. The completed 20 μl cDNA syntheses were diluted to 50 μl with nuclease-free water and stored at −20°C. This template cDNA was diluted 2-fold prior to use, and 1 μl of the resulting solution was used as template for qPCR. The PCRs were conducted on an Applied Biosystems (Foster City, CA USA) 7300 Real-Time PCR System using 25-μl preparations that consisted of 12.5 ul Go-Tag Colorless Master Mix (Promega, Madison, WI, USA), 1 μl cDNA, 200 μM each primer, 0.85X SYBR Green I (Invitrogen, Carlsbad, CA, USA), and 300 nM ROX dye (Roche, Indianapolis, IN, USA) as a passive fluorescence standard. The qPCR software available with the instrument was used to determine the Ct, the fractional cycle number at which the fluorescence intensity reached an arbitrary threshold, using the threshold determined by the software. Relative fold change was determined using the delta-delta Ct method [19]. Expression levels of three individual genes encoding calcium-dependent protein kinases (GenBank accessions AY280366, AY332386 and DQ333376) were found to be unchanged by the temperature treatments (S1 Fig) and were used as multiple, constant-expression housekeeping genes as described by Vandesompele et. al [20].

Expression levels were assessed with qPCR of six GenBank accessions annotated as being from genes encoding chlorophyll a/b binding proteins (accessions BQ172277, BQ620062, CK164231, CK211804, CK212505 and U73218), accession CA731337, encoding cinnamoyl-CoA reductase, and accession JX679079, encoding WRKY80 transcription factor. Determinations were carried out with three technical replicates of each of two biological replicates of each gene.

## Results

**Fig 1:**
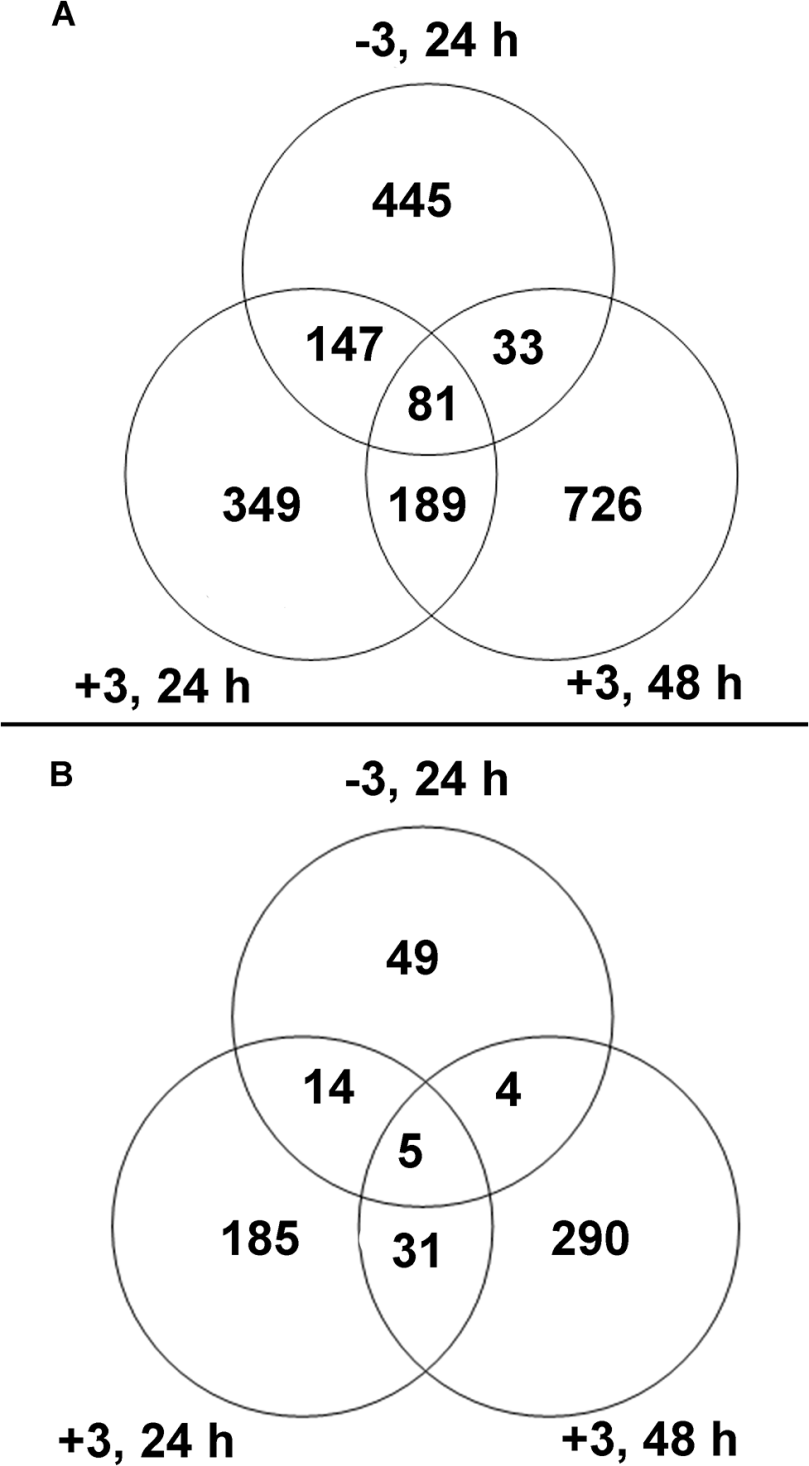
Numbers of genes in wheat crowns changing expression after exposure to the indicated temperature treatments. (**A**) Numbers of upregulated genes, (**B**) numbers of downregulated genes. All plants treated were exposed to −3°C for 24 h; plants from the +3, 24 h and +3, 48 h treatments were subsequently allowed to thaw at +3°C for 24 or 48 h, respectively, after the freeze.

A total of 1970 genes were more than 2-fold upregulated (P< 0.05) in response to one or more of the temperature treatments, relative to the control (Fig 1A). In contrast, only 578 genes were significantly downregulated at any of the time points examined relative to the control, and only five of those genes were downregulated throughout the 72 h. freeze-thaw process (Fig 1B; S1 Table).

Thirty-four GO terms were over-represented in common after each of the three freeze/thaw treatments; 68 GO terms were uniquely over-represented after the plants had been exposed to −3°C for 24 h; 22 were uniquely over-represented after the plants had subsequently thawed at +3°C for 24 h; and 119 were uniquely over-represented after the plants had thawed at +3°C for 48 h (S2 Table).

**Fig 2:**
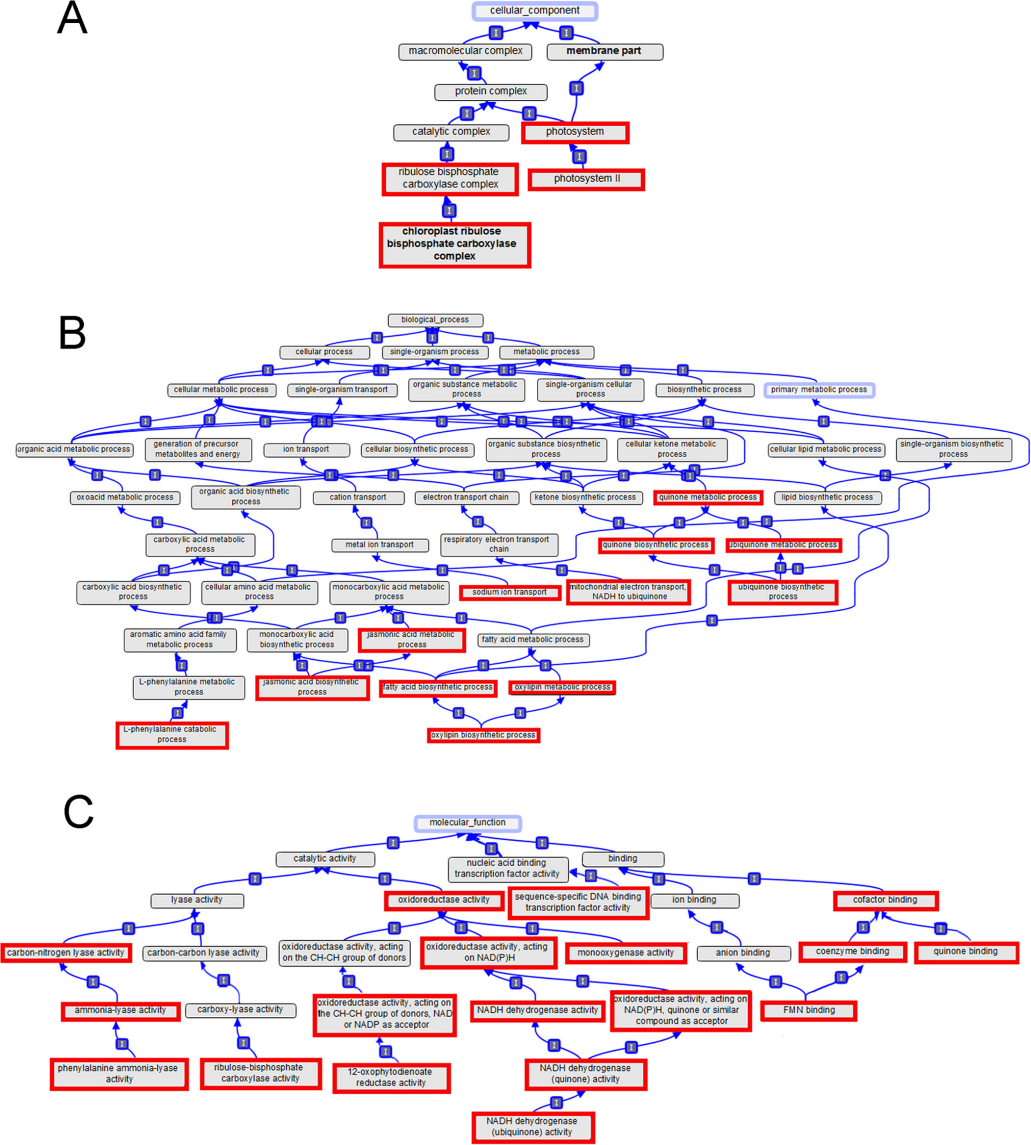
Gene ontology diagrams showing the 34 GO terms significantly responding to freeze-thaw in wheat crowns. Plants were: frozen to −3°C for 24 h; frozen to −3°C for 24 h then allowed to thaw at +3°C for 24 h; frozen to −3°C for 24 h then allowed to thaw at +3°C for 48 h. The GO terms outlined with red were significantly over-represented in common after each of the three temperature treatments. Terms are shown from three GO domains (**A:** cellular component, **B:** biological process, and **C:** molecular function).

The 34 GO terms that were over-represented in common at each of the time points comprised four terms from the cellular component ontology, 12 from the biological process ontology, and 18 from the molecular function ontology. The four from the cellular component ontology were all related to chloroplast function, including photosystem II and ribulose bisphosphate carboxylase (Rubisco) function (Fig 2A). The 12 GO terms from the biological process ontology were consistent with increased respiration and stress response (Fig 2B). Five GO terms were related to quinone/ubiquinone processing (Fig 2B), suggesting enhanced functioning of the mitochondrial respiratory electron transport chain. Concomitantly, Na^+^ ion transport function was enhanced; Na^+^ ion concentration influences respiration efficiency in complex ways [21,22]. Go terms related to oxylipin metabolism, including the oxylipin jasmonic acid, also were over-represented (Fig 2B). Oxylipins play a prominent role in cell signaling in plant stress response [23]; the over-representation of these terms suggested the 24h exposure to −3°C activated stress response mechanisms that continued to be expressed at high levels for at least 48 h after the subzero temperature stress was removed. The L-phenylalanine catabolism process also was over-represented at each of the time points (Fig 2B). Phenylalanine can be catabolized to fumarate and then contribute to respiration via the citric acid cycle, and phenylalanine also can be catabolized to trans-cinnamic acid by phenylalanine ammonia lyase activity and entered into the phenylpropanoid biosynthetic pathway.

Phenylalanine ammonia lyase activity was one of the 18 molecular function GO terms that were over-represented at each of the three time points (Fig 2C), suggesting the phenylpropanoid biosynthetic pathway was raised to a higher level of activity after the plants were exposed to −3°C for 24 h, and this enhanced activity continued for at least 48 h after the subzero temperature stress was removed.

In addition to these 34 GO terms significantly over-represented at each of the time points, there were 29 biological process GO terms over-represented only after 24 h at −3°C (S2 Table). A GO survey similar to Fig 2 revealed these over-represented terms referred to metabolic processes including “alkaloid biosynthetic process” and “amine catabolism”; energy-related processes including “mitochondrial electron transport, cytochrome c to oxygen” and “ribosome biogenesis”; transcription and DNA replication-related processes including “DNA unwinding involved in DNA replication” and “transcription, DNA-templated”; and stress response processes including “defense response, incompatible interaction,” “response to salicylic acid,” and “salicylic acid mediated signaling pathway”. Genes representing “proton transport” also were significantly over-represented (S2 Table). Proton transport is vital to numerous processes [24], consistent with the concept that exposure of the plants to −3°C resulted in the initiation of numerous responses.

There also were 10 molecular function GO terms uniquely over-represented after the −3°C freezing phase (S2 Table). These included binding to: phospholipids, NADP, RNA, or Ca++; and “acid-ammonia (or amide) ligase,” “nucleotidyltransferase activity,” and “structural constituent of ribosome.”

The cellular component GO terms that were uniquely over-represented after the −3°C freezing phase (S2 Table) indicated that the responses involved nearly all cellular components including mitochondria, ribosomes, nuclear chromosomes, plastids, and “organelle subcompartment” and “organelle lumen.”

After the sub-zero temperature stress was removed, only 8 biological process GO terms, and 11 molecular function terms were significantly over-represented uniquely after the plants had been allowed to thaw at +3°C for 24 h (S2 Table), suggesting a small number of processes in addition to those initially upregulated during the −3°C freeze (Fig 2) were upregulated during the first 24 h of thawing. In contrast, 35 molecular function and 81 biological process GO terms were significantly over-represented after the plants had remained at +3°C for an additional 24 h (−3, 24h, then +3, 48 h, S2 Table). This large difference in over-represented GO terms at the 24 h and 48 h post-freezing time points indicated the −3°C, 24 h freeze initiated a response that proceeded with relatively narrow scope during the freeze and within the first 24 h of thawing at +3°C, then greatly diversified during the 24–48 h post-freezing period.

The 34 GO terms that were over-represented in common among the temperature treatments (Fig 2) were represented by a number of probesets (putative genes) at each of the three time points. There were 255, 245, and 320 probesets that contributed to the 34 GO terms at the −3°C, 24h, +3°C, 24h, and +3°C, 48h time points, respectively, (probesets not shown). This observation suggested that several genes of similar function, and thus with the same gene ontology annotation, may have been upregulated at one of the time points, but not necessarily at another time point. Also, while 23% of the individual genes that responded significantly to the temperature treatments were down-regulated (Fig 1, S1 Table), no GO terms associated with down-regulated genes were significantly over-represented at any of the time points examined. This result suggested that down-regulation occurred with essentially equal frequency across the functions and processes represented by the GO terms. Thus, it appeared that precise regulation of specific genes encoding similar functions, rather than regulation of categories of genes during the freeze-thaw process was involved in conditioning the resulting enhanced freezing tolerance.

Of the 1970 individual genes upregulated at least 2-fold, 1520 (77%) were upregulated in response to only one of the temperature treatments (Fig 1A), suggesting that the response to the freeze/thaw treatment was essentially sequential on an individual gene basis, with different genes responding during the subfreezing temperature phase and other genes responding in sequential fashion after the plants had been returned to +3°C. Consistent with the over-representation of GO terms (S2 Table), nearly twice as many genes were uniquely upregulated after 48 hours of thawing compared to either of the other temperature treatments (Fig 1A), suggesting processes underway by the end of 48 h of thawing were quite distinct from those that were active during the −3°C freeze or the first 24 h of thawing. To investigate this phenomenon further, the frequencies of individual genes that represented broad categories of gene function and had been uniquely upregulated in only one of the temperature treatments were compared.

**Fig 3:**
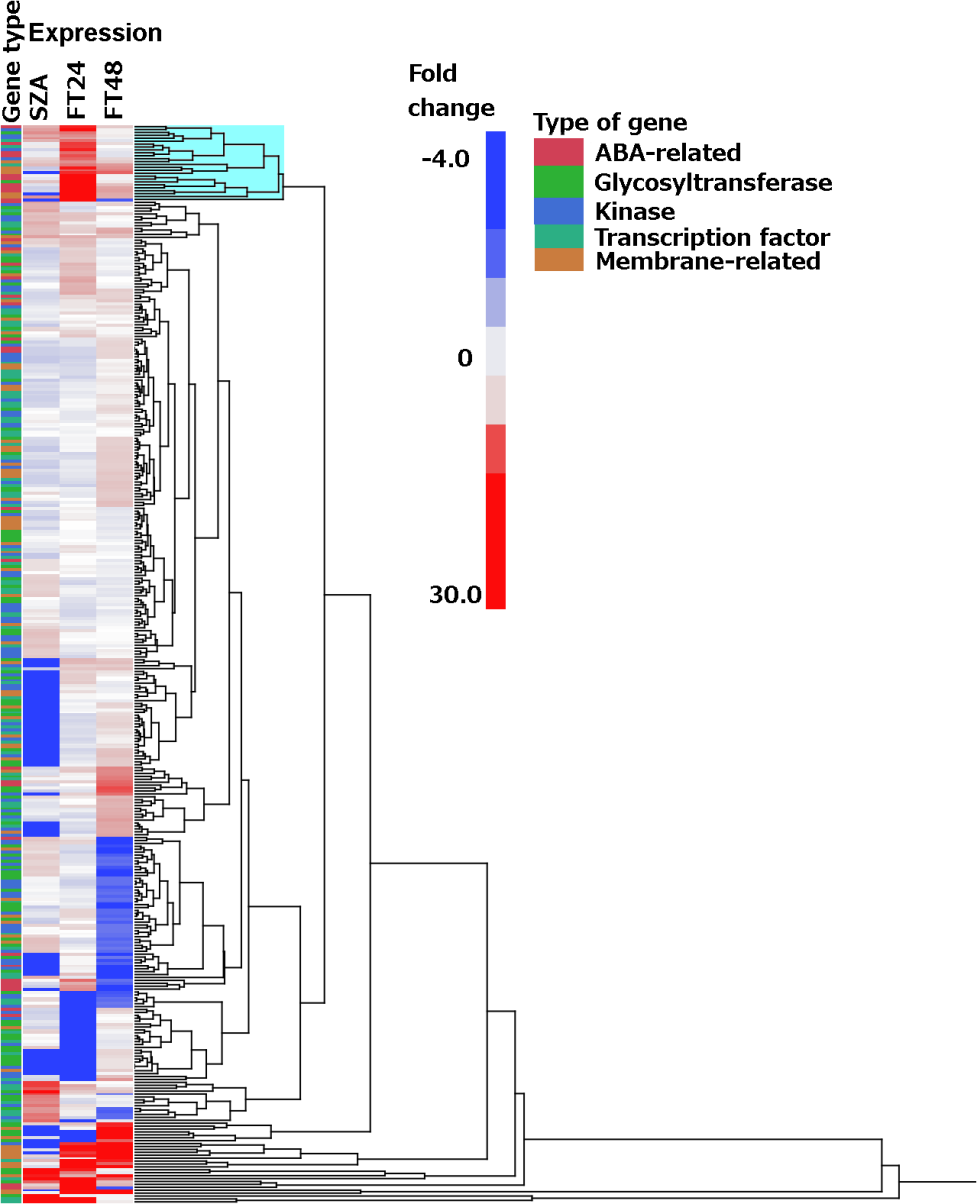
Expression level responses to freeze-thaw treatments of five classes of genes in wheat crown tissue. All genes shown were upregulated at least 2-fold in at least one of the treatments. SZA: subzero acclimation, −3°C for 24 hours; FT24: freeze/thaw 24 hours, frozen to −3°C for 24 hours then allowed to thaw at +3°C for 24 hours; FT48: freeze/thaw 48 hours, frozen to −3°C for 24 hours then allowed to thaw at +3°C for 48 hours. The cluster of genes highlighted in blue at the top of the figure were uniquely, strongly upregulated only in response to the FT24 treatment (see text for details).

No significant homology with any genes of known function was found for 480 of the 1,970 genes that were upregulated at least 2-fold and therefore these genes remain of unknown function. All others were manually assigned to a broad functional category according to the descriptions available as shown in S1 Table; examples are shown in Fig 3.

**Fig 4:**
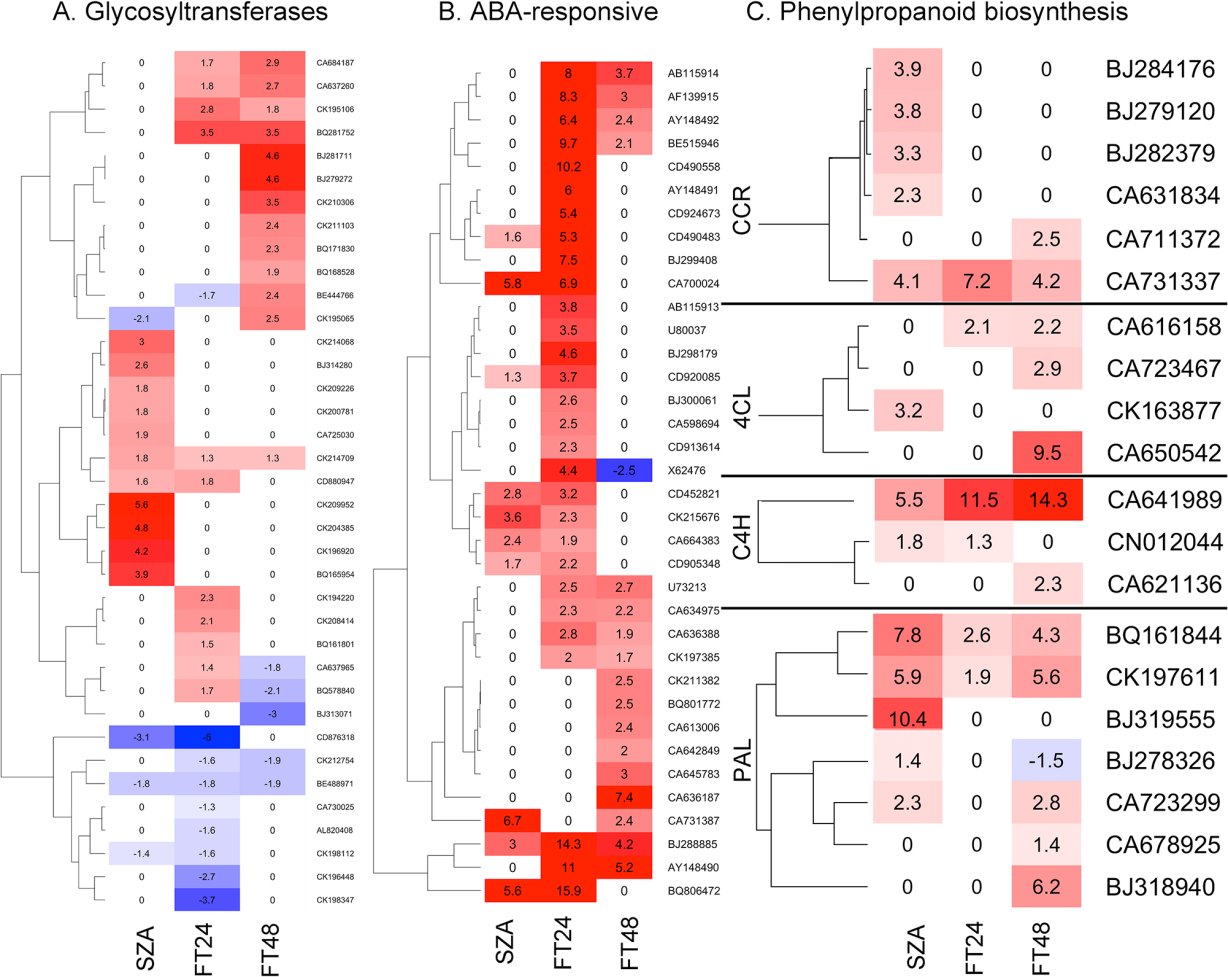
Expression level changes of three classes of genes in wheat crowns following three freezing treatments. SZA: subzero acclimation, −3°C for 24 hours; FT24: freeze/thaw 24 hours, frozen to −3°C for 24 hours then allowed to thaw at +3°C for 24 hours; FT48: freeze/thaw 48 hours, frozen to −3°C for 24 hours then allowed to thaw at +3°C for 48 hours. Responding genes encoding enzymes of the phenylpropanoid biosynthetic pathway were phenylalanine ammonia lyase (PAL), cinnamate-4-hydroxylase (C4H), coumarate-CoA ligase (4CL), and cinnamoyl-CoA reductase (CCR). Expression level fold-changes relative to the control (cold-acclimated plants) are indicated; levels indicated as zero were not significantly different from the control.

Many genes were broadly classified as abscisic acid (ABA)-responsive, glycosyltransferases, kinases, transcription factors, or as encoding membrane-related proteins. Clustering of these genes on the basis of expression level changes relative to the control revealed that several cohorts of genes responded in characteristic, sequential fashion over the 72 h time course examined. For example, 37 genes encoding glycosyltransferase activity were significantly up- or down-regulated during the 72 h time course (Fig 4A). A group of 11 of these genes were significantly upregulated after 24 h. at −3°C; nine of these had been downregulated by the end of the subsequent thaw at +3°C for 24 h., but a different group of nine genes had been upregulated during the thaw, resulting in 11 genes being upregulated after the first 24 h. of thawing (Fig 4A). Six of the 11 genes in this second group had been downregulated after an additional 24 h. at +3°C, but a third group of eight genes had been upregulated such that 13 genes encoding glycosyltransferase activity were expressed to significantly higher levels than the control after freezing, then thawing for 48 h. (Fig 4A). Expression levels of another nine genes encoding glycosyltransferase activity were either unchanged or downregulated during the freeze, then were downregulated further during the first 24 h of thawing, and remained downregulated during the second 24 h of thawing or returned to pre-freezing levels of expression (Fig 4A). These results suggested that exposure to −3°C for 24 h activated processes that continued to develop and diversify long after the temperature returned to +3°C, further suggesting that many of the processes active in each time interval were distinct from the processes active in the other time intervals, including processes involving glycosylation. Genes upregulated during the −3°C, 24 h time interval were upregulated in advance of the other time intervals studied and therefore may have been instrumental in initiating later processes.

The phytohormone ABA is involved in response to numerous stress factors and both ABA–dependent and ABA–independent pathways have been shown to be involved in cold temperature response [25]. Of 36 significantly upregulated ABA-responsive genes identified in this study, 10 were upregulated during the −3°C freeze; 9 of these 10 remained upregulated during the first 24 h of thawing, but all had returned to pre-freezing levels of expression by the end of the second 24 h of thawing, with a single exception (Accession BJ288885, Fig 4B). During the first 24 h of thawing at +3°C, 20 additional ABA-responsive genes were upregulated, but 11 of those were downregulated to pre-freeze expression levels by the end of the second 24 h of thawing (Fig 4B). Seven additional ABA-responsive genes were significantly upregulated between 24 and 48 h of thawing (Fig 4B). These results are consistent with involvement of ABA in the initiation and propagation of some of the processes involved in freeze/thaw enhanced freezing tolerance, often this involvement is conditioned by specific genes upregulated for a relatively short period of time.

The phenylpropanoid biosynthetic pathway is responsible for the generation of numerous secondary metabolites and is involved in the response of plants to biotic [26] and abiotic [27] stresses. The initial part of this pathway includes the sequential function of phenylalanine ammonia lyase (PAL), cinnamate-4-hydroxylase (C4H), coumarate-CoA ligase (4CL), and cinnamoyl-CoA reductase (CCR). The expression levels of genes encoding these four enzymes showed significant upregulation in response to the −3°C freeze, followed by downregulation of some of these genes, but also upregulation of other genes encoding similar function during the following 24–48 h of thawing (Fig 4C).

These indications from transcriptional responses of glycosyltransferases, ABA-responsive genes, and the phenylpropanoid biosynthetic pathway again indicated that the response to the −3°C, 24 h freeze was primarily the initiation of processes that continued and diversified after the subzero temperature stress had been removed. Expression level dynamics of many other classes of genes followed a similar pattern (S1 Table). These genes included numerous transcription factors and Zn-containing proteins (potentially also transcription factors). Genes encoding C-repeat binding factor (CBF) transcription factors are shown separately because of their extensive involvement in response to cold temperature [28, 29]. Along with the transcription factors, several genes encoding proteins involved in calcium trafficking were uniquely upregulated, proportionally more after 24 h at −3°C than after thawing (S1 Table). The responsiveness of these genes, along with the large number of kinases that also were upregulated (S1 Table) is consistent with very active cell signaling processes, suggesting gene activity during the 24 h, −3°C period was instrumental in triggering downstream processes that manifested over 24–48 h at +3°C, leading to enhanced freezing tolerance.

During the first 24 h of thawing, 20 genes encoding proteins identified as localizing to the membrane were uniquely upregulated (S1 Table). This observation suggests that the response to the −3°C, 24 h freeze had progressed to the point of physical modification of the cell by the end of the first 24 hours after the temperature had returned to + 3°C.

By the end of the second 24 h of thawing at +3°C, proportionally more genes potentially involved in physical modification of the cell had been upregulated than during the other two time periods (S1 Table). Numerous genes encoding proteins involved in molecular binding, molecular transport, carbohydrate metabolism, and oxidation/reduction regulation; and genes encoding transferases and signal protein molecules were all significantly, uniquely upregulated (S1 Table), again consistent with the indication of many biological processes becoming active during 24–48 h of thawing (S1 Table), suggesting a broad range of adaptive responses.

**Fig 5:**
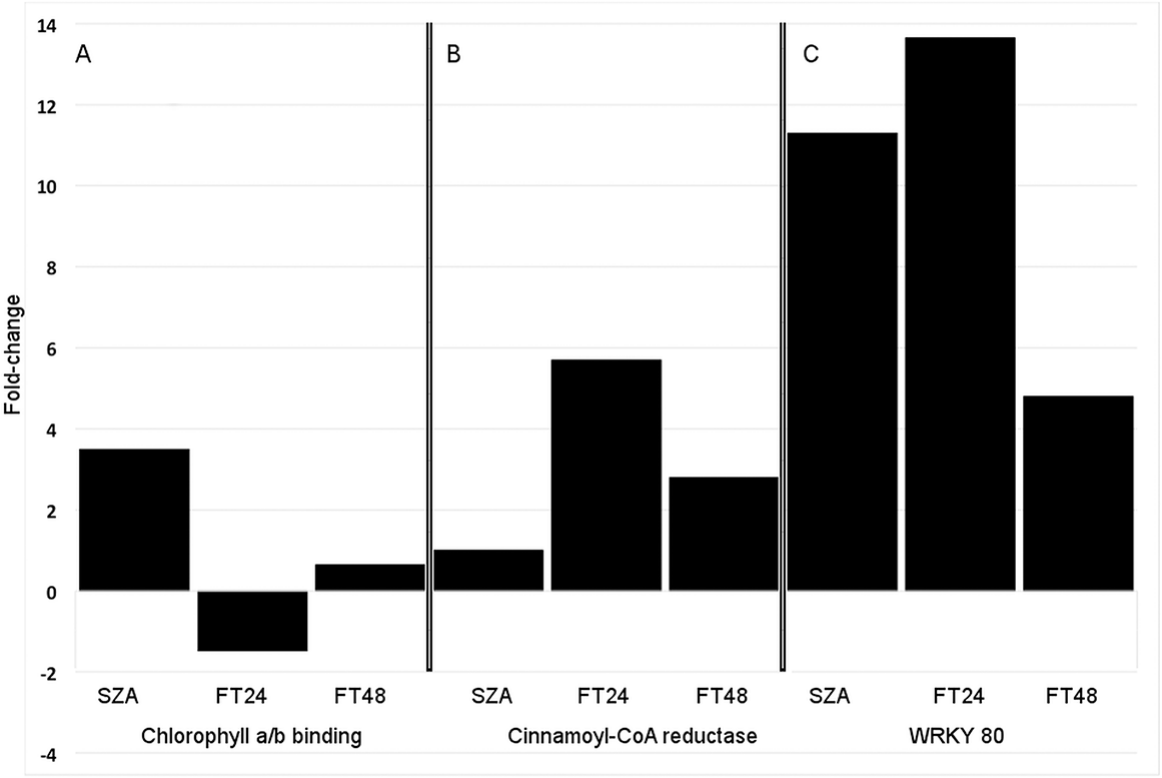
Expression level fold-changes of three classes of genes in wheat crowns following freeze-thaw treatments. Fold changes were determined with quantitative real-time PCR. SZA: subzero acclimation, −3°C for 24 hours; FT24: freeze/thaw 24 hours, frozen to −3°C for 24 hours then allowed to thaw at +3°C for 24 hours; FT48: freeze/thaw 48 hours, frozen to −3°C for 24 hours then allowed to thaw at +3°C for 48 hours. **(A)** Chlorophyll a/b binding gene expression fold-change shown represents the mean of six genes. **(B)** Cinnamoyl-CoA reductase is a key gene of the phenylpropanoid biosynthetic pathway. **(C)** transcription factor WRKY80.

The expression levels of many of these genes appeared to be strongly responsive to changes in temperature, sometimes with subsequent amelioration of the response with continued exposure to that same temperature. For example, most of the cluster of 26 genes highlighted in blue at the top of Fig 3 responded little to the onset of subfreezing temperature (−3°C), but then were strongly upregulated within 24 h after the freezing stress was removed and the plants were returned to +3°C. However, after the plants had been maintained at +3°C for an additional 24 h, the expression levels of these genes were decreased to near initial levels (Fig 3). Many additional examples of genes that responded strongly in only one of the time periods examined were evident (Fig 3). This kind of expression control was confirmed with qPCR on five genes encoding proteins homologous to chlorophyll a/b binding proteins. We previously demonstrated that genes apparently encoding chlorophyll a/b binding proteins were significantly upregulated as the temperature was slowly decreased to potentially damaging levels [30]. The five genes examined in this study were very similarly regulated; the average response of the five genes is shown in Fig 5. Consistent with the microarray results (Fig 4C), a gene encoding cinnamoyl-CoA reductase was shown to have been upregulated after exposure to subfreezing temperature, more strongly upregulated after thawing at +3°C for 24 h, then somewhat downregulated after thawing for an additional 24 h, but still expressed to a higher level than the control (Fig 5). We previously demonstrated that several WRKY transcription factors responded strongly to potentially damaging subfreezing temperatures [30] and in the present study, expression of WRKY80 (GenBank accession JX679079) was increased more than 10-fold after exposure to −3°C, upregulated nearly 14-fold after thawing at +3°C for 24 h, then downregulated during the next 24 h of thawing, but still remained more than 4-fold upregulated relative to the control (Fig 5). In each of these examples, the removal of the freezing stress resulted in a significant change in expression within 24 h, followed by further change while at a constant temperature.

## Discussion

Freezing fully cold-acclimated wheat plants to −3°C initiates a process that ultimately leads to enhanced tolerance of subsequent freezing to potentially damaging temperatures [10, 11]. This effect is further enhanced if the plants are allowed to thaw at a low, positive temperature for 24–48 h before freezing to potentially damaging temperatures [11]. This study indicated that significant upregulation of many genes (Fig 1), involved in many biological processes and molecular functions (Fig 2; S2 Table) is associated with this enhancement of freezing tolerance. Many of these processes and functions were upregulated in sequential fashion (S2 Table), and most (77%) of the upregulated individual genes remained upregulated for only about 24 h (Fig 1), suggesting a progression of the freezing tolerance enhancement process through a series of functions encoded by a series of genes. Many classes of gene function were represented in each time period studied, but often, this gene function was represented by different individual genes within each time period (Figs 3 and 4). This phenomenon was most apparent with genes encoding glycosyltransferase and kinase activity, which together accounted for about 10% of the genes uniquely upregulated in each time period (S1 Table). The glycosyltransferase gene family in plants is very large [31–33], as is the kinase gene family [34], hence it is not surprising that individual glycosyltransferase and kinase genes may be considerably specialized.

The glycosyltransferase genes encode a very diverse array of related activities, such that these enzymes can be classified into at least 92 families [35]. Numerous glycosyltransferases interact with specific acceptor molecules including secondary metabolites and hormones [35]. Thus, the unique upregulation of numerous glycosyltransferase genes in each of the phases of the freeze/thaw treatment (Fig 4A) is consistent with their involvement in sequential progression of a response initiated during the −3°C freeze that continued to develop and diversify throughout the 48 h of thawing at +3°C. Prior evidence that glycosyltransferase activity may be essential to diversification of ongoing stress response processes was found in a study of salt stress response in tobacco (*Nicotiana tabaccum*) plants ectopically expressing a gene from *Arabidopsis thaliana* encoding a glycosyltransferase [36]. Not only did the transgenic plants have improved salinity stress tolerance, but they also exhibited extensive remodeling of the transcriptome following exposure to the salt stress compared to non-transformed control plants [36]. This finding indicated that the glycosyltransferase activity was integral to cell signaling and propagation of the processes that led to improved salinity stress tolerance. In the present study, the observation that many glycosyltransferases were uniquely upregulated at the time points examined (Fig 4A) may have similarly indicated that specific glycosyltransferase activities were required sequentially for the propagation of the processes that led to improved freezing tolerance.

Kinases may phosphorylate multiple target molecules and target molecules may be phosphorylated by multiple kinases, hence multiple pathways may be tied together into networks by kinases [37, 38]. There is ample evidence of complex signaling networks related to stress response in plants. For example, the expression patterns of genes responding to cold, drought, salinity and ABA application overlap, suggesting cross-talk of the involved signaling pathways [25, 39], possibly mediated by kinases. In the present study, 105 probesets representing kinases showed specific transcriptional regulation responses (S1 Table), indicating active involvement in each of the phases of the freeze-thaw process.

The phenylpropanoid biosynthetic pathway appeared to have been upregulated throughout each phase of the freeze/thaw cycle (Fig 2C), and glycosyltransferases that modify the products of the phenylpropanoid biosynthetic pathway are known [31, 35]. Thus, it appears that one part of the response to freeze-thaw stress may include upregulation of the phenylpropanoid biosynthetic pathway and further modification of the secondary metabolites produced by that pathway.

Literally thousands of secondary metabolites are generated from the phenylpropanoid biosynthetic pathway products and their subsequent modification [40, 41]. How these metabolites may contribute to increased freezing tolerance is not known, although a correlation of the production of anthocyanin (a product of the phenylpropanoid biosynthetic pathway) and cold acclimation of grasses (among other species) has been known for many years [42]. Furthermore, several genes involved in the phenylpropanoid biosynthetic pathway were shown to be upregulated in freezing tolerant, but not freezing sensitive tomato lines after exposure to low temperature [43], and in a broad-ranging survey, it was reported that phenylpropanoid metabolism may be involved in response to at least seven different kinds of stress in *Arabidopsis thaliana* including cold stress [44]. These observations suggest that the phenylpropanoid biosynthetic pathway may play a significant role in cold tolerance of many plant species and the results reported here suggest that in cold-acclimated wheat plants, these kinds of responses are initiated in response to subzero temperatures and continue to be expressed after the freezing stress is removed.

Similarly, many genes involved in calcium trafficking were upregulated in each phase of the freeze/thaw cycle; proportionally more during the −3°C freeze (S1 Table), suggesting that calcium trafficking was instrumental in initiating and sustaining this stress response. It is known that ice crystals form within wheat crown tissue at about −3°C [45] and as extracellular ice forms, the concentration of dissolved solutes in the extracellular fluid is effectively increased as liquid water migrates out of the cells in response to the induced chemical gradient [28]. Thus, the intracellular concentration of the ions present, including Ca^++^ is effectively increased at −3°C. Ca^++^ is a very active signal molecule involved in all aspects of plant growth and development [46] and numerous Ca^++^ responsive genes were upregulated throughout the freeze-thaw process (S1 Table), again consistent with the concept that numerous metabolic pathways become involved in the freezing tolerance responses initiated by freezing to −3°C.

There may be thousands of plant genes that respond primarily or secondarily to ABA [47], and ABA can function as a long-distance signal molecule [48], thus, the strong upregulation of ABA-responsive genes during the first 24 h of thawing (Fig 4B) may have been consistent with the initiation of multiple stress responses that were not activated at subzero temperatures. There also were proportionally more genes encoding membrane proteins upregulated during thawing at +3°C than during freezing at −3°C, suggesting processes leading to physical remodeling of the cell membrane had been triggered by the 24 h subzero exposure and these processes continued after the freezing stress had been removed. Structural modifications also were implicated in response to −3°C in *Arabidopsis thaliana* [49].

By the end of 48 h of thawing at +3°C, proportionally more genes involved in stress response and possibly physical remodeling had been upregulated, including genes encoding proteins involved in redox balance, carbohydrate metabolism, and molecular binding, transferase, and transport activities. Thus, one interpretation of these results is that the freeze-thaw process as described here appeared to initiate a cell signaling process that led to activation of stress response including both ABA-dependent and independent mechanisms, and these, in turn, led to membrane and metabolite modifications that result in improved ability to withstand subsequent freezing to potentially damaging temperatures. The observation that many more biological processes and molecular functions had been uniquely enhanced after 48 h of thawing compared to the other two time points (S2 Table) suggests that numerous processes that would continue to develop for days had been set in motion by the single 24 h freeze to −3°C.

## Conclusions

This study showed that the improved freezing tolerance of wheat plants that results from exposure to −3°C [10, 11], and subsequent thawing at +3°C [11] appears to result from the activation of numerous biological processes and depends on the adequate functioning of many genes, some of which may be active for only a brief period of time during freezing stress response and recovery. The necessity for these multiple processes probably accounts for the commonly observed over-dispersion of wheat freezing survival data such that zero-inflated statistical models more accurately predict survival [50], indicating multiple points of failure can occur during the development of freezing tolerance. We previously demonstrated [30] that the expression levels of hundreds of genes were significantly upregulated in cold-acclimated winter wheat plants as the temperature decreased to potentially damaging levels, and that plants with differing freezing tolerance potentials regulated expression of specific genes differently at low, potentially damaging temperatures. Hence, identifying plant lines that are especially effective in activating parts of the response to −3/+3°C freeze-thaw described here, and plants especially effective in responding to potentially damaging temperatures, may provide plant sources that can be genetically combined to yield greater freezing tolerance and winterhardiness.

## Supporting Information

S1 Fig Amplification plot of a housekeeping gene. Real-time PCR amplification plot of one of three calcium-dependent protein kinases used as housekeeping genes; GeneBank accession number: DQ333376.
(TIF)

S1 Table Microarray probesets showing significant up- or down-regulation in wheat crown tissue following exposure to mild freezing. (XLSX)

S2 Table Significantly over-represented gene ontology terms found in transcriptomic expression study of wheat crown tissue following exposure to mild freezing. (XLS)
