# Supplementary figures and images for: Genes Upregulated in Winter Wheat (Triticum aestivum L.) during Mild Freezing and Subsequent Thawing Suggest Sequential Activation of Multiple Response Mechanisms
Source: PLoS One. 2015 Jul 14;10(7):e0133166. doi: 10.1371/journal.pone.0133166 (PMC4501828; doi:10.1371/journal.pone.0133166)

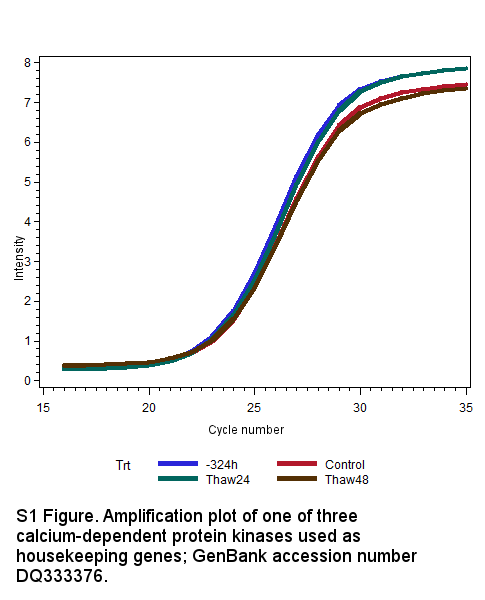

Supplement: S1 Fig — Real-time PCR amplification plot of one of three calcium-dependent protein kinases used as housekeeping genes; GeneBank accession number: DQ333376. (TIF) [file pone.0133166.s001.tif]
